# Supplementary material for: Identification of photoperiod-induced specific miRNAs in the adrenal glands of Sunite sheep (Ovis aries)
Source: Front Vet Sci. 2022 Jul 22;9:888207. doi: 10.3389/fvets.2022.888207 (PMC9354845; doi:10.3389/fvets.2022.888207)
Supplement: Supplementary file 1 [file Data_Sheet_1.ZIP › Supplementary file/miRDeep2 file.zip!/miRDeep2 file/Novel_Structure/LP42A2/Novel_391.pdf]

Provisional ID	: NC_019464.2_15104
Score total	: 0
Score for star read(s)	: -1.3
Score for read counts	: -2.3
Score for mfe	: 2
Score for randfold	: 1.6
Score for cons. seed	:
Total read count	: 7
Mature read count	: 7
Loop read count	: 0
Star read count	: 0

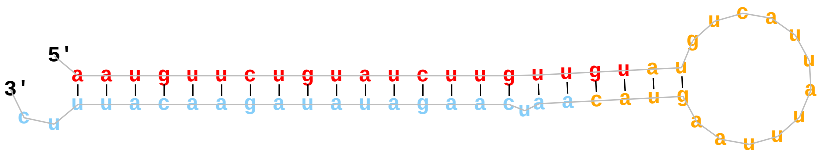

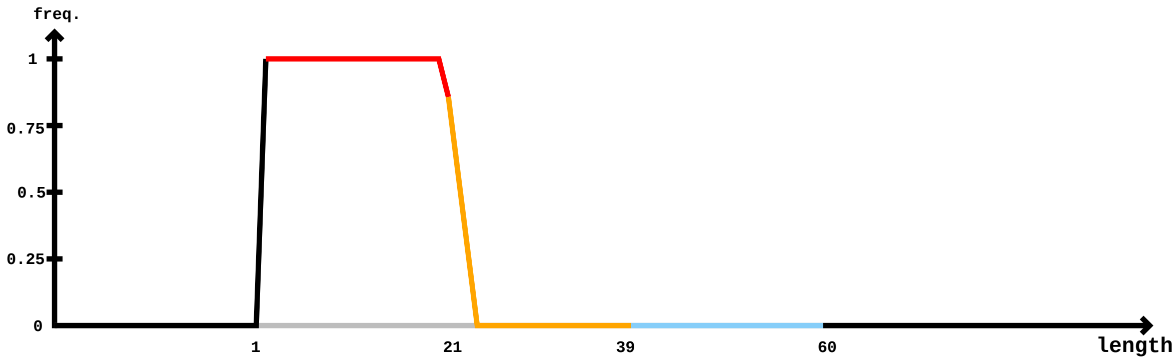

Mature **Star**

```
5'- ggaaaccuggggguaauggaaauguucuguaucuuguuguaugucauuauuuaaguacaaucaagauauagaacauuuccauuacacucaagcuuaauuaaauggauauaug   -3'    exp
    .......((((.((((((((((((((((((((((((((((((............)))))).)))))))))))))))))))))))).))))......................    reads      mm         sample
    ....................aauguucuguaucuuguug.........................................................................    1          0          seq
    ....................aauguucuguaucuuguugu.......................................................................    4          0          seq
    ....................aauguucuguaucuuguuguau......................................................................    2          0          seq
```
